# Mimicking climate warming effects on Alaskan soil microbial communities via gradual temperature increase

**DOI:** 10.1038/s41598-020-65329-x

**Published:** 2020-05-22

**Authors:** Max-Bernhard Ballhausen, Rebecca Hewitt, Matthias C. Rillig

**Affiliations:** 1 Freie Universität Berlin, Institut für Biologie, Plant Ecology, Berlin, Germany; 2 Berlin-Brandenburg Institute of Advanced Biodiversity Research (BBIB), Berlin, Germany; 3 Center for Ecosystem Science and Society, Northern Arizona University, Flagstaff, USA

**Keywords:** Microbial ecology, Soil microbiology

## Abstract

Climate change can trigger shifts in community structure and may therefore pose a severe threat to soil microbial communities, especially in high northern latitudes such as the Arctic. Arctic soils are covered by snow and ice throughout most of the year. This insulation shields them from high temperature variability and low surface temperatures. If this protective layer thaws, these soils are predicted to warm up at 1.5x to 4x the rate of other terrestrial biomes. In this study, we sampled arctic soils from sites with different elevations in Alaska, incubated them for 5 months with a simulated, gradual or abrupt temperature increase of +5 °C, and compared bacterial and fungal community compositions after the incubation. We hypothesized that the microbial communities would not significantly change with a gradual temperature treatment, whereas an abrupt temperature increase would decrease microbial diversity and shift community composition. The only differences in community composition that we observed were, however, related to the two elevations. The abrupt and gradual temperature increase treatments did not change the microbial community composition as compared to the control indicating resistance of the microbial community to changes in temperature. This points to the potential importance of microbial dormancy and resting stages in the formation of a “buffer” against elevated temperatures. Microbial resting stages might heavily contribute to microbial biomass and thus drive the responsiveness of arctic ecosystems to climate change.

## Introduction

Climate change is the defining issue of our time^1^ and is perceived as the most serious problem affecting the world today (Global Shapers Survey, World Economic Forum 2017). On a global scale, temperatures have been rising at an alarming pace since the age of industrialization. The last IPCC Synthesis report^2^ predicted a further increase of 1.5 °C to 5 °C in the next 100 years. Although all parts of the planet will be affected by climate change, the Arctic has been and is going to be more severely affected than the global average. In the last decade, the poles have been warming at a rate that is more than the double of the global average warming, a phenomenon that has been called “Arctic Amplification”^3,4^. The mechanism is probably a decrease in albedo caused by the retreat of melting snow and ice pack. With an ambient temperature increase, the exposed earth surface would not be “shielded” by a reflective layer of snow and ice and absorb more sunlight which would accelerate heating of the surface, thus creating a high relative temperature increase.

The arctic covers an area of ~12 million km² of which ~5 million km², an area of about half the size of the United States or China, is mostly vegetated by tundra with a foundation of permafrost. The topsoil of arctic tundra ecosystems is vegetated by graminoids, mosses, lichens, and small shrubs. The zone underneath the active soil layer, called permafrost, is permanently frozen and impenetrable to plants and animals. Conservation of permafrost soils is of pivotal importance for the stability of the global climate because they store large amounts of carbon. This carbon, if released into the atmosphere via thawing of permafrost and/or accelerated decomposition, could double the CO_2_ in the earth’s atmosphere^5^. The topsoil in most parts of the artic is frozen for two thirds of the year and maximum summer soil temperatures, depending on elevation, can be as low as 8 °C. The low temperature, short growing season, and low nutrient input naturally constrain decomposition of organic matter. The high relative temperature increase (8 °C to 12 °C) together with a prolonged summer season due to earlier snow melt in spring and later freezing in autumn would be expected to increase microbial activity and decomposition, and thus elevate the CO_2_ release from arctic soils.

Microbial life in the arctic is adapted to harsh conditions. Even though the short growing season leaves only a short time window for reproduction, fungi in this biome have a long lifespan and slow population growth, which results in lower population turnover as compared to more temperate climates^6^. It has, however, been reported that the slow and steady fungal growth can produce 10 times more fungal than bacterial biomass in arctic soils. Fungal activity even seems to peak when the soil is covered by snow^7^ and growth has even been observed in frozen soils with a temperature as low as −2 °C^8^. Arctic soils consist of layered plant material (leaves, twigs, roots) in various stages of decomposition, on top of permafrost soil or bedrock. Since decomposition is slow, small amounts of steadily accumulating plant material can form organic layers of up to 1 m thickness^9^.Fungi catalyse key steps in decomposition of organic matter, are involved in the cycling of nearly all plant nutrients, and therefore contribute significantly to arctic ecosystem functioning. Since fungi are suspected to be vulnerable towards a changing climate, they received considerable research attention in polar regions. The latest reports indicate a richness of up to 4350 different fungal Operational Taxonomic Units (OTUs) in the entire arctic^6,10^. Surprisingly, for arctic regions only low endemism has been reported. Saprotrophic fungal diversity has been shown to be similar along latitudes with the same taxonomical groups occurring even at the earths two distant poles, the arctic and Antarctica^11^. The reason for this is probably extensive aerial spore dispersal via clouds or as part of fine dust winds that move air masses through the earth’s atmosphere^12,13^. A similar trend has been observed for soil bacterial diversity and community composition. The most dominant artic bacterial phyla, similar to soils of lower latitudes, are Actinobacteria, Bacteriodetes, Acidobacteria, Alphaproteobacteria, and Betaproteobacteria. Similar to lower latitudes, the distribution of bacteria in the arctic can be best explained by pH rather than geographic proximity of sites suggesting that environmental factors and not dispersal limitation as a driving force for shaping fungal communities in the arctic^14^.

The diversity and abundance of macroorganisms is negatively correlated with elevation. This relationship is can be monotonous decline or humped back. By contrast, the diversity of microorganisms like bacteria or fungi does not seem to follow a predictable pattern or trend. Microbial diversity has been shown to be negatively correlated with elevation^15^, positively influenced by elevation^16^, and to rather be dependent on abiotic soil parameters like pH or snow cover, or biotic factors like plant community composition^17,18^. To our knowledge, there is only one study that looks at microbial abundance and community shifts along an altitudinal gradient in the arctic. In their study, Kotas *et al*.^19^ find no significant shifts in abundance or community composition of fungi or bacteria associated with elevation.

It makes intuitive sense to apply a gradual than an abrupt temperature increase if one would want to mimic climate change experimentally. All the climate change scenarios that are used to predict climate change in the latest IPCC report, however, implicitly assume a gradual rather than an abrupt increase in temperature. Nevertheless, almost all climate change studies apply an abrupt temperature increase treatment. Studies that compare rates of change or contrast an abrupt versus a gradual treatment find variable results on different organisational levels (physiological response of a single organism, population responses of multiple individuals, shifts in a microbial community composition, or shifts in nutrient fluxes on the ecosystem scale^20–23^). Abrupt stress application directly triggers a physiological stress response which should in the most extreme scenario lead to death of individuals or population and a resulting shift in community composition and finally ecosystem functioning. If a stress factor is gradually applied, acclimation in the short-, and adaptation in the long term is possible. This would result in less severe shifts in microbial community composition and ultimately stabilized ecosystem functioning.

In our study, we sampled arctic soils from two different elevations (Toolik Field Station at 759 m and Chandelar Shelf at 1000 m), shipped them to the lab, and tested the effect of a +5 °C temperature increase on soil fungal and bacterial communities. The temperature treatment was applied in a gradual and in an abrupt way over a total incubation time of ca. 5 months (154 days). At the end of the experiment we determined the bacterial and fungal community composition and measured decomposition as an ecosystem function.

We hypothesized that we would observe a shift in microbial community composition and a drop in microbial diversity since the applied temperature treatment is a relatively high temperature as compared to the low baseline temperatures that Alaskan soils experience. We also expected a shift in ecosystem functioning, namely an increase in decomposition, caused by the elevated temperatures applied. We expected that abruptly applied temperature treatments would shift microbial community compositions, whereas gradually increasing temperatures would allow for acclimation and adaptation and would thus not cause major community changes.

## Material and Methods

### Soil sampling

We sampled soils at two sites along an elevation gradient on the north side of the Brooks Range (one site north of Toolik Field Station and one south of Toolik Field Station, called “Chandalar Shelf”). Each site was located off the Dalton Highway/Haul Rd to Prudhoe Bay. At each site, we navigated to a point located at the target elevation within 1 km of the Dalton HWY. We haphazardly selected the starting point of a 50 m transect running due north. We harvested the top ten centimeters of soil at five locations ten meters apart along the transect. Each of these locations represents one replicate for each of the sites. Vegetation was trimmed from the top of the sample. Soils were placed in a cooler and kept cold but not frozen. Soils were sampled on August 21 (Toolik Field Station) and August 22 (Chandalar Shelf), shipped on August 23rd, and delivered to Berlin (Germany) on August 25th, 2017. Upon arrival, soils were frozen at-20 °C until the start of the experiment. Soils were homogenized with a blender and stones were removed. The experiment was set-up in 50 ml falcon tubes and since the amount of organic matter varied substantially between sites, tubes were filled by volume rather than weight. Every tube received about 20 ml of soil. Water content of the samples was determined and adjusted to 65% of the maximum water holding capacity (WHC) with sterile water. The water content was checked and adjusted to 65% WHC on a regular basis throughout the experiment.

### Decomposition treatment

Half of the samples received wooden beech wood sticks as a common decomposition substrate. The wood was autoclaved, dried at 60 °C till constant mass, and incubated fully submerged in the soil in the 50 ml tubes. At the end of the experiment wooden sticks were cleaned by removing attached soil using a brush and dried at 60 °C to a constant mass. Afterwards mass loss during the experiment was calculated.

### Warming treatments

To simulate climate change experimentally, we applied different warming treatments over a total period of 5 months. We chose a site-specific baseline temperature which was the maximum temperature that the Chandalar Shelf and Toolik Field Station experienced in the year 2015 and 2016, respectively (temperature data downloaded from https://permafrost.gi.alaska.edu and https://toolik.alaska.edu/). The incubation was started at the baseline temperature (TS = 19 °C, CS = 7.5 °C) which was applied for the first 42 days of the experiment, a timeframe that we considered sufficient for the development of a stable microbial community before the application of the climate change treatments. Afterwards the temperature was either abruptly (in a few minutes), or gradually increased (0.045 °C/day for 90 days) to the maximum temperature which was 5 °C higher than the baseline temperature (TS = 24 °C, CS = 12.5 °C), a temperature increase which corresponds to the maximum amount of temperature increase predicted in the IPCC report^2^. In order to keep the absolute amount of temperature/energy that the warming treatments received the same (the area under the curve, i.e. the same temperature-days), the microcosms that received the abrupt temperature treatments were harvested 22 days earlier than the gradual and control treatment.

### Molecular methods

Upon harvest, the soil was homogenized thoroughly, and DNA was isolated in triplicates from microcosms without wooden sticks. Microcosms with wooden sticks were only used to determine weight loss through decomposition and excluded from molecular analyses. The extractions were done with the DNEASY Powersoil HTP 96 Kit (Qiagen, Hilden, Germany) according to the manufacturer’s instructions. DNA was eluted in 100 µl water. DNA extraction was confirmed with loading 10 µl of extracted DNA on a 1.5% agarose gel. The homogenized pool of the three independent DNA isolations served as the template for fungi and bacteria specific PCRs. To amplify bacteria, per sample, six independent PCR reactions consisting of 16.25 µl water, 5 µl KAPA HiFi Fidelity Buffer, 0.75 µl dNTPs, 0.75 µl of primers 515 f and 806r targeting theV4 region of the 16 S rDNA, 0.5 µl KAPA HiFi DNA Polymerase, and 1 µl template DNA were run in a 25 µl reaction volume with the following conditions: 95 °C for 30 sec, followed by 30 steps of 98 °C for 30 sec, 55 °C for 30 sec, and 72 °C for 30 sec, and finally a elongation at 72 °C for 5 min. Fungal PCRs were done with the same reagents and concentrations except the primers used were ITS4 and ITS7 targeting the ITS region on the fungal rDNA. The following cycling conditions were used: 95 °C for 5 min, 30 cycles of 98 °C for 20 sec, 55 °C for 30 sec, and 72 °C for 30 sec, and finally 72 °C for 2 min. PCR products were examined on a 1.5% agarose gel and subsequently cleaned from residual primers and pooled with a SPRI magnetic bead cleanup protocol. Briefly, PCR products (6 × 24 µl) were mixed in a 1 to 0.8 ratio with AMPure XP magnetic beads (Beckman Coulter, High Wycombe, UK). Incubated for 5 min at room temperature and placed for 5 min on a magnetic plate. The supernatant was removed, and the beads were washed twice with 70% EtOH. Finally, the beads were air dried for 10 min, re-dissolved in 25 µl sterile water, incubated for 5 min at room temperature, placed back on the magnetic plate for 2 min, and the supernatant was transferred to a new plate. Removal of primers was confirmed via 1.5% agarose gel. In the next step, indexing PCRs were carried out, adding sample specific barcodes to the PCR products. Indexing PCRs were run in 25 µ, in duplicates, with the following conditions: 95 °C for 3 min, 8 steps of 98 °C for 30 sec, 55 °C for 30 sec, and 72 °C for 30 sec, with a final elongation at 72 °C for 5 min. Reactions consisted of 14.8 ul water, 5 µl KAPA HiFi Fidelity Buffer, 0.75 µl dNTPs, 0.75 µl of forward and reverse indexing primers (Supporting material table 1), 0.5 µl KAPA HiFi DNA Polymerase, and 8 µl template DNA.

The indexing PCR was confirmed on a 1.5% agarose gel and residual primers were removed with SPRI beads (see above). Absence of primers was confirmed on a 1.5% agarose gel, and the DNA concentration was measured via a Picogreen assay (Quant-iT PicoGreen dsDNA Kit, Thermo Fisher Scientific, Waltham, USA) according to the manufacturer’s instructions. Fluorescence was measured against a standard curve with a FLUOstar OPTIMA plate reader (BMG Labtech, Ortenberg, Germany). Libraries of barcoded samples were prepared via equimolar pooling according to DNA concentrations determined by the Picogreen assay. Absence of primers was finally confirmed via automated system gel electrophoresis, Tapestation D-1000 (Agilent, Waldbronn, Germany) and a D1000 Screen Tape. The concentration of the library was determined with a Qubit together with the Qubit dsDNA HS kit (Thermo Fisher Scientific, Waltham, USA), diluted to a concentration of 5 ng/µl, and finally sequenced at the Berlin Center for Genomics in Biodiversity Research (BeGenDiv, Berlin, Germany) on the MiSeq system, using v3 chemistry (2 × 300 paired-end reads).

### Bioinformatics and statistics

Data were analysed using the Qiime2 pipeline^24^. Briefly, primers and adapters were removed using “cutadapt”, “dada2” was used to denoise reads, to join paired-end reads, and to finally create Exact Sequence Variants (ESVs). Forward and reverse reads for both organism groups were truncated from base 272 and 203 onwards, respectively. Taxonomy was assigned using the machine learning classifier “sklearn”, trained on version 7.2 of the unite database for fungi^25^ and version 13.8 of the greengenes database^26^ for bacteria, in both cases using at 99% threshold for clustering. Fungal and bacterial sequences were merged, exported into.biom format and imported for statistical analysis into R. In R, we used the package “phyloseq”^27^ to analyse the dataset. Alpha diversity was calculated using the Shannon index and rarefaction curves were calculated with the function ggrare, using a non-truncated dataset in both cases. For follow up analyses, singletons were removed, and samples were rarefied to the lowest number of reads which was 5000 reads for the fungal, and 21179 reads for the bacterial dataset. To visualize variation in community composition among treatments and elevations we calculated NMDS ordinations based on relative abundance data and Bray Curtis distances. Fungal and bacterial diversity was estimated using the “Shannon” index and compared between elevations using a t-test after homogeneity of variance was confirmed by using Levene’s test. We used multivariate analysis of variance (PERMANOVA, function adonis, 999 permutations) to test for differences between elevations (759 m and 1000 m) and treatments (abrupt, gradual, control). The dispersion in each of the groups (elevations and treatments) as a measure of intra-group variability was tested using BETADISPERSER, function permutest, 999 permutations. Amplicon sequencing data was deposited at the European Nucleotide Archive (ENA), accession number PRJEB34523.

Effects on decomposition were investigated with a two-way ANOVA with elevation (759 m and 1000 m) and treatment (gradual, abrupt, or no temperature increase) as factors. Data were log-transformed, and homogeneity of variance was confirmed by Levene’s test.

## Results

After quality filtering and trimming, we retrieved a total of 1,129,311 fungal, and 1,535,896 bacterial high-quality reads. This translated into a total of 2,888 fungal and 19,298 bacterial ESVs. Rarefaction curves for bacteria and fungi showed saturation and thus confirmed that we did not underestimate the diversity of either group (Supplementary Figures 1 and 2).

**Figure 1:**
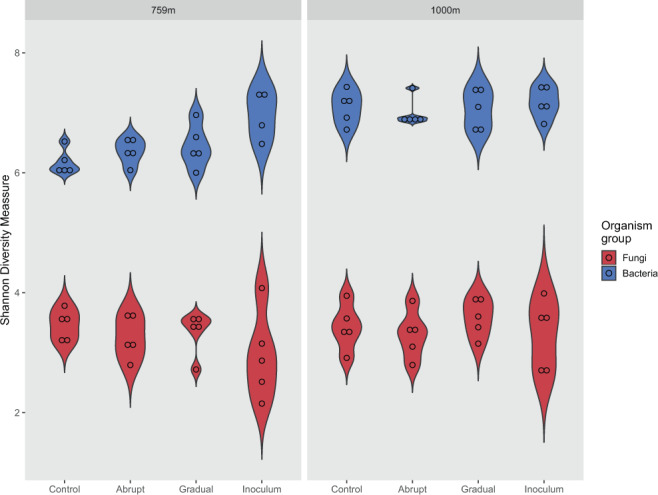
Violin plots of Shannon Diversity Indices of the fungal (red) and bacterial (blue) communities from 759 m and 1000 m elevation. “Control”, “abrupt”, “gradual”, and “inoculum” communities are compared. Inoculum refers to the non-incubated starting community. Violins show the density distribution of the data.

Bacterial diversity was lower at 1000 m as compared to 759 m elevation for “abrupt”, “gradual”, and “control” temperature treatments (p = 0.002, p = 0.023, and p = 0.001, respectively). There was no difference between the bacterial starting communities (inocula) and also not between the fungal treatments/inocula (Fig. 1).

**Figure 2:**
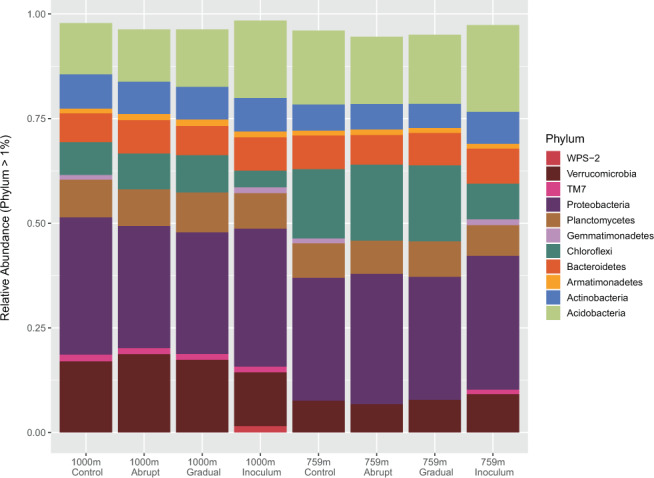
Bacterial phyla compared between elevations 759 m and 1000 m and between experimental treatments (“abrupt”, “gradual”, “control”, and “inoculum”. Inoculum refers to the non-incubated starting community. Only phyla with a relative abundance higher than 1% are displayed.

Concerning bacteria, overall, the most dominant phyla (>2% abundance) were: Proteobacteria (31%), Acidobacteria (16%), Verrucomicrobia (12%), Chloroflexi (12%), Planctomycetes (8%), Bacteroidetes (8%), and Actionbacteria (7%). We observed a shift in Chloroflexi and Verrucomicrobia that was associated to elevation. Verrucomicrobia increased (8% to 17%) and Chloroflexi decreased (15% to 8%) with elevation (p = 0.019 and p = 0.037) (Fig. 2).

**Figure 3:**
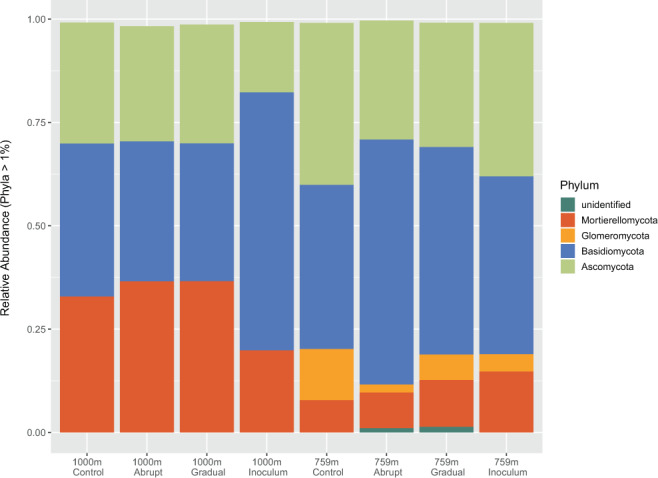
Fungal phyla compared between elevations 759 m and 1000 m and between experimental treatments (“abrupt”, “gradual”, “control”, and “inoculum”. Inoculum refers to the non-incubated starting community. Only phyla with a relative abundance higher than 1% are displayed.

The most abundant fungal phyla in our dataset were Basidiomycota (44%), Ascomycota (29%), Mortierellomycota (22%), and Glomeromycota (3%) (Fig. 2). The fungal communities between 759 m and 1000 m differed mainly because of the absence of the Glomeromycota from the higher elevations (Fig. 3).

**Figure 4:**
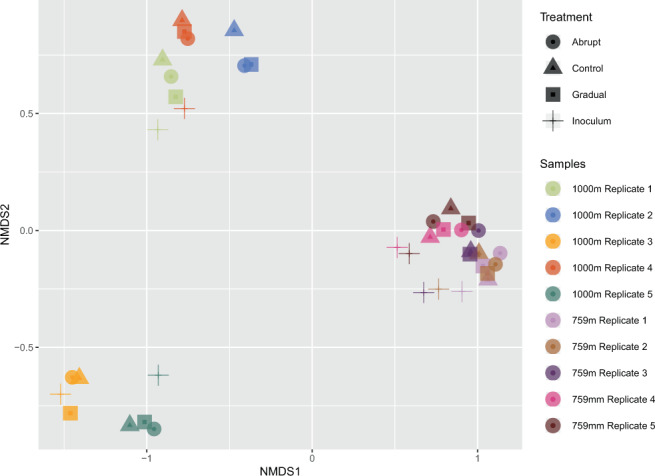
NMDS ordination plot of the bacterial community compositions. Shapes show the experimental treatments (circles = “abrupt”, triangles = “control”, squares = “gradual”, crosses = “inoculum”). Colours indicate independent biological replicates.

**Figure 5:**
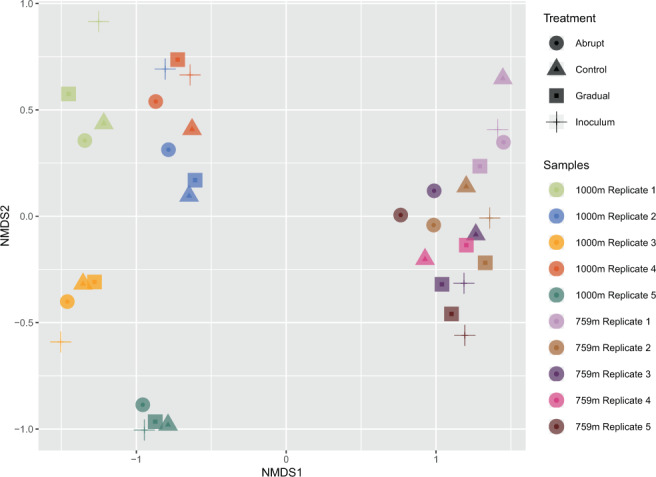
NMDS ordination plot of the fungal community compositions. Shapes show the experimental treatments (circles = “abrupt”, triangles = “control”, squares = “gradual”, crosses = “inoculum”). Colours indicate independent biological replicates.

In both organism groups, bacteria and fungi, communities differed between 759 m and 1000 m elevation (PERMANOVA, F = 14.293, R² = 0.27865, p < 0.001; Figs. 4 and 5, respectively). Bacterial and fungal communities also differed concerning the dispersion inside the elevational groups with higher elevations being more variable (BETADISPERSER, F = 56.817, p = 0.001). We did not find differences between communities treated with an abrupt, gradual, or no (control) temperature increase. There was, however, a difference between the bacterial communities from 759 m elevation: starting community (inoculum) and control (F = 1.5871, R² = 0.16555, p = 0.02, 999 permutations); inoculum and abrupt (F = 1.5871, R² = 0.16555, p = 0.02, 999 permutations); and inoculum and gradual (F = 1.9592, R² = 0.19672, p = 0.009, 999 permutations)(Fig. 3).

**Figure 6:**
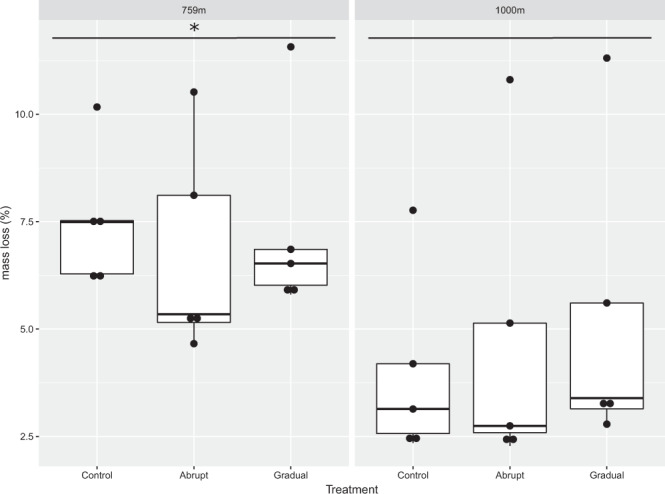
Boxplot graph of observed decomposition during the experiment. Mass loss (g) that occurred during the incubation of samples with “abrupt”, “gradual”, or “control” incubation temperature, contrasted between the two elevations (759 m and 1000 m). Significant difference between the elevations is depicted with a star.

We found an overall difference in decomposition between the two elevations (F = 11.384, p = 0.00255) with 759 m elevation having a higher decomposition rate than 1000 m elevation. We found no differences between the treatments (gradual, abrupt, control) within the elevations. Average mass loss was 7,22%(standard deviation 2,07) and 4,65%(standard deviation 3,00) from 759 m and 1000 m samples, respectively (Fig. 6).

## Discussion

We incubated Alaskan soils that had been sampled at two different altitudes (759 m and 1000 m) for 5 months at elevated temperatures (+5 °C), simulating an abrupt and a gradual temperature increase. We found bacterial and fungal communities to be different between elevations, independent of the temperature treatments.

It was mainly the absence of the fungal phylum Glomeromycota in the 1000 m samples as compared to a relative overall abundance of about 6% in the 759 m samples that differentiated the fungal communities between the two elevations (Fig. 1). Glomeromycota are mycorrhizal fungi that are obligate biotrophs which live in symbiosis with a host plant. They colonize host roots, enhance the uptake of plant nutrients like phosphorus and nitrogen, and get plant derived carbon in return. It has been shown before that the abundance of arbuscular mycorrhizal fungi (AMF) decreases with elevation with differing patterns between AMF genera^28^. Since AMF have been routinely found at elevations of 1000 m and higher, we assume that the absence of AMF at the higher elevation here is not related to altitude per se but can rather be explained by the absence of a suitable host plant.

Bacterial phyla that made the difference between 759 m and 1000 m elevation were the Verrucomicrobia, which increased from 8% to 17%, and the Chloroflexi, which decreased from 15% to 8% between 759 m and 1000 m, respectively (Fig. 2). There is only one study that looked at Verrucomicrobia community composition along an elevational gradient. In that study^29^, the authors find correlations between the different classes of Verrucomicrobia, pH and/or soil carbon to nitrogen ratio, but no difference in the abundance of Verrucomicrobia in soils from different elevations. Verrucomicrobia are known to be oligotrophic and very sensitive to disturbance^30^. Their presence is often negatively correlated to physically disturbing agricultural practices like soil tillage, for example^31^. It would seem reasonable that Verrucomicrobia are also vulnerable to the harsher conditions that come with higher elevation but instead we can report on the opposite. It is not clear why Verrucomicrobia in the arctic would be less sensitive to disturbance. We conclude that more data are needed to derive any general pattern on the abundance of Verrucomicrobia in arctic or tundra soils. Chloroflexi are commonly found in arctic soil or sediment communities^32^. Tundra soil might even be one of the habitats with the highest diversity of Chloroflexi^33^. Our results confirm the finding of another study that finds higher Chloroflexi abundance to be related with elevation^34^. Another study in northern Alaska found high Chloroflexi abundance to be related to the depth of snow coverage^35^. Higher elevations receive higher amounts of snow and the snow melts later in the year. The increased abundance can thus be decoupled from elevation and is probably a result of reduced oxygen availability and increased soil temperatures underneath an increased snowpack^35^.

Although studies have reported contrasting results concerning bacterial and fungal diversity along elevational gradients, the general trend goes towards a decline of species richness with an increase in elevation. This is because of a general increase in environmental “harshness” rather than any predictable variation in abiotic or biotic factors^16,18^. We also find a decrease in species richness with elevation for the bacterial but not for the fungal dataset. While bacterial diversity was higher at 759 m as compared to 1000 m (Fig. 3), fungal diversity stayed the same over the two sampling points (Fig. 4). This is in accordance with a recent study by Ren *et al*.^36^ who found that the diversity of bacterial but not of fungal communities responded to differences in elevation on the Qinling Mountains in eastern mainland China. The authors propose that bacterial communities were more prone to react to elevation specific differences in abiotic factors and shifts in the plant community as compared to the fungal communities. Another explanation for this could be fungal ability to form spores or sclerotia. These structures allow fungi to overcome stressful conditions and disperse to new habitats that can be many hundreds of kilometres away^13^. When sequencing the soil microbial communities, we did not differentiate between spores and mycelium, hence, we might have partly been comparing spore derived fungal communities. Once deposited, fungal spores can remain viable but inactive for several years. Harsh climatic conditions favour high sporulation, coupled with long dormancy and low spore decay. Therefore, arctic soils can harbour relatively big spore banks. Sequence data from those soils might have been derived from dormant or even dead spores which would have masked differences between the active fungal communities from the sampled elevations in our dataset.

Although the fungal diversities between elevations were not different, the fungal community from the lower elevation was more active than the community from higher elevation and able to decompose about 38% more substrate during the experiment (Fig. 5). The absolute mass loss was, however, very low (about 4% of the initial weight was decomposed). This is in contrast to decomposition rates of about 40% for similar substrates in temperate grassland ecosystems^37^. Therefore, we conclude that soil fungal activity, in general, is low, which is also reflected in the lack of apparent differences in decomposition among the temperature treatments.

Surprisingly, there was no detectable shift in community composition between the warming treatments (control, gradual, abrupt) for either of the groups, fungi or bacteria (Figs. 4 and 5). This is in stark contrast to the hypothesis that Alaskan soils would be vulnerable to a changing climate because of the high temperature increase (+5 °C) relative to the low ambient temperature. Our study is not the first to discover low responsiveness of high latitude soil microbes to climate change. Rinnan *et al*. reported in^38^ on a 15 year experiment that applied warming and fertilization treatments to field plots in the arctic. They found that only after simulating climate change for 15 years, differences between microbial diversity or abundance between warming and control became apparent. In another field warming experiment, Allison *et al*.^39^ found no shift in fungal community composition or activity in terms of CO2 production even after having the experiment run for 7 years. They did, however, find an increase in chitin- and cellulose degrading enzymes with elevated temperatures^40^. Darrouzet-Nardi *et al*. recently, in^41^, performed a 3 year warming experiment which was realized with the use of open-top-chambers, simulating an earlier thawing of the soil and elevated soil temperatures throughout the season. They found no effects on microbial activity, plant root growth or biomass.

The reasons for the low responsiveness of arctic microbial systems to elevated temperature could lie in the potentially large spore bank. We could find no information about the spore bank of saprotrophic fungi in the arctic. There is, however, one study on the AMF spore bank in arctic meadows by Varga *et al*.^42^. The authors find that arctic AMF spore banks are a) larger than temperate spore banks b) contain spores with a similar viability (~30%) compared to temperate spore banks and c) require specific cues, probably for a longer duration than in temperate systems, to break spore dormancy. In case of saprotrophic fungi and bacteria, microbial activity in the arctic might be very much dependent on nutrient input. Specific nutrient cues are probably necessary to break dormancy of fungal spores and activity of bacteria is down regulated if nutrients are scarce. Low nutrient availability would foster an oligotrophic microbial community with an inherently low responsiveness to climate change treatments. We sampled the soils at the end of the growing season, in late August, right before the onset of the first snowfall. Possibly, the sampled community had already gone through an adjustment towards lower nutrient availability and formation of resting stages. Shifts in substrate usage between “summer” and “winter” has been shown for alpine soils. In winter season microbial life would be sustained via decomposition and the breakdown of organic polymers and phenols, whereas in summer root exudates would become the major carbon source^43^. The late summer community would already have undergone sporulation and most of its fungal and bacterial members would have entered a “resting stage”, preparing for the winter. Even though arctic soils have been shown to harbour a very active winter community, large parts of this community might be dormant and unresponsive until exposure to the necessary cues. This could have implications for fungal community turnover as well as functional responsiveness such as decomposition. Microbial communities in winter are very stable in terms of species composition but since they arctic winter is relatively long the accumulated loss of CO_2_ from soil can be very high. Natali *et al*.^44^ applied only slightly elevated temperatures (+1.5 °C) in an arctic field experiment during winter. While this did not lead to an increase in summer respiration, they found the winter microbial communities to respire twice as much CO_2_ as compared to the control treatment. Even though we were freezing the soil for a few weeks after the sampling, in order to simulate the winter freezing, this period of frost was relatively short and was, together with the lack of nutrients after thawing, perhaps not enough to activate dormant microbes and make them respond to the climate change treatments. Even though arctic microbial communities are adapted to low nutrient inputs and exert rather low metabolic activity, we cannot exclude the possibility that our microcosms ran out of easily available nutrients. This would have restricted microbial community turnover and could be an alternative explanation for the non-observed shifts in community structure.

Finally, we can conclude that the simulated climate change treatments did not affect the microbial community composition of Alaskan soils. We set the maximum temperature that those soils experienced during a hot summer day as the baseline (control) for the incubations and hypothesized that an elevated temperature of +5 °C on top of these temperatures would change community compositions. In our experiment, microbial communities were not as responsive to a simulated climate change than we predicted. This could have either been because of limitations in our experiment e.g. we were not able to break the dormancy of the microbial community because the applied freezing period was too short, we incubated a late summer microbial community which just went dormant, or because of biological reasons, meaning that arctic microbes are indeed better prepared for temperature changes than we thought. Many studies that report on an elevated respiration rates or dramatic changes in arctic microbial communities as a response to a simulated temperature increase involve warming of permafrost soils. Those soils have been frozen for long periods of time and have completely different microbial communities that react more sensitively to thawing than the topsoil that we investigated here. Those are not permanently frozen and instead exposed to freezing only on an annual basis.

For this study, we sampled organic surface layers of soil which mainly consist of plant derived organic material in various stages of decomposition. These organic surface layers cover deeper permafrost layers, which underlie around 38% of the Alaskan mainland^45^. The layers can “buffer” fluctuating climate such as temperatures and protect the underlying permafrost. A stable fungal community in these surface layers permits continuous decomposition of organic matter and formation of soil organic carbon while preventing the permafrost from thawing, which would induce a loss of soil carbon. Compared to other biomes such as the tropics or subtropics, soil carbon in the arctic is stored mainly in the organic surface and the permafrost layers and not so much in the aboveground plant biomass. Although the vulnerability of permafrost has been shown in other studies, our study proposes that in soils on top of the permafrost, microbial decomposition of organic surface layers might be resistant to global warming and therefore possibly provide a “buffer” to permafrost temperature increases.

Since several long-term field experiments, in agreement with our study, were not able to find an effect of simulated climate change on microbial community composition in arctic ecosystems, we do not think that longer term experiments are needed to more realistically investigate climate change experimentally. Instead, we propose to investigate temperature effects on microbial resting stages like spores, sclerotia, and dormant cells separately from the active biomass/fungal mycelium^46^. Fungal spores could for example be isolated from soil and DNA could be extracted separately from mycelial and spore compartments. Afterwards, microbial communities could be analysed from both fractions and responses to temperature treatments could be investigated separately. Another option would be to compare 16SrRNA and 16SrDNA communities, the former indicating the active microbial community whereas the latter would also include inactive organisms that do not actively synthesize RNA^47^. Future studies would also profit from microbial respiration data. This would make conclusions about microbial activity shifts which do not per se entail community shifts possible.

## Supplementary information

Supplementary information.
