# Supplementary material for: Mimicking climate warming effects on Alaskan soil microbial communities via gradual temperature increase
Source: Sci Rep. 2020 May 22;10:8533. doi: 10.1038/s41598-020-65329-x (PMC7244726; doi:10.1038/s41598-020-65329-x)
Supplement: Supplementary file 1 — Supplementary information. [file 41598_2020_65329_MOESM1_ESM.pdf]

Supplementary material

Mimicking climate warming effects on Alaskan soil microbial communities via gradual temperature increase

Authors

Max-Bernhard Ballhausen[1,2,*], Rebecca Hewitt[3], Matthias C. Rillig[1,2]

Affiliation

[1] Freie Universität Berlin, Institut für Biologie, Plant Ecology, Berlin, Germany

[2] Berlin-Brandenburg Institute of Advanced Biodiversity Research, Berlin, Germany

[3] Center for Ecosystem Science and Society, Northern Arizona University, Flagstaff, USA

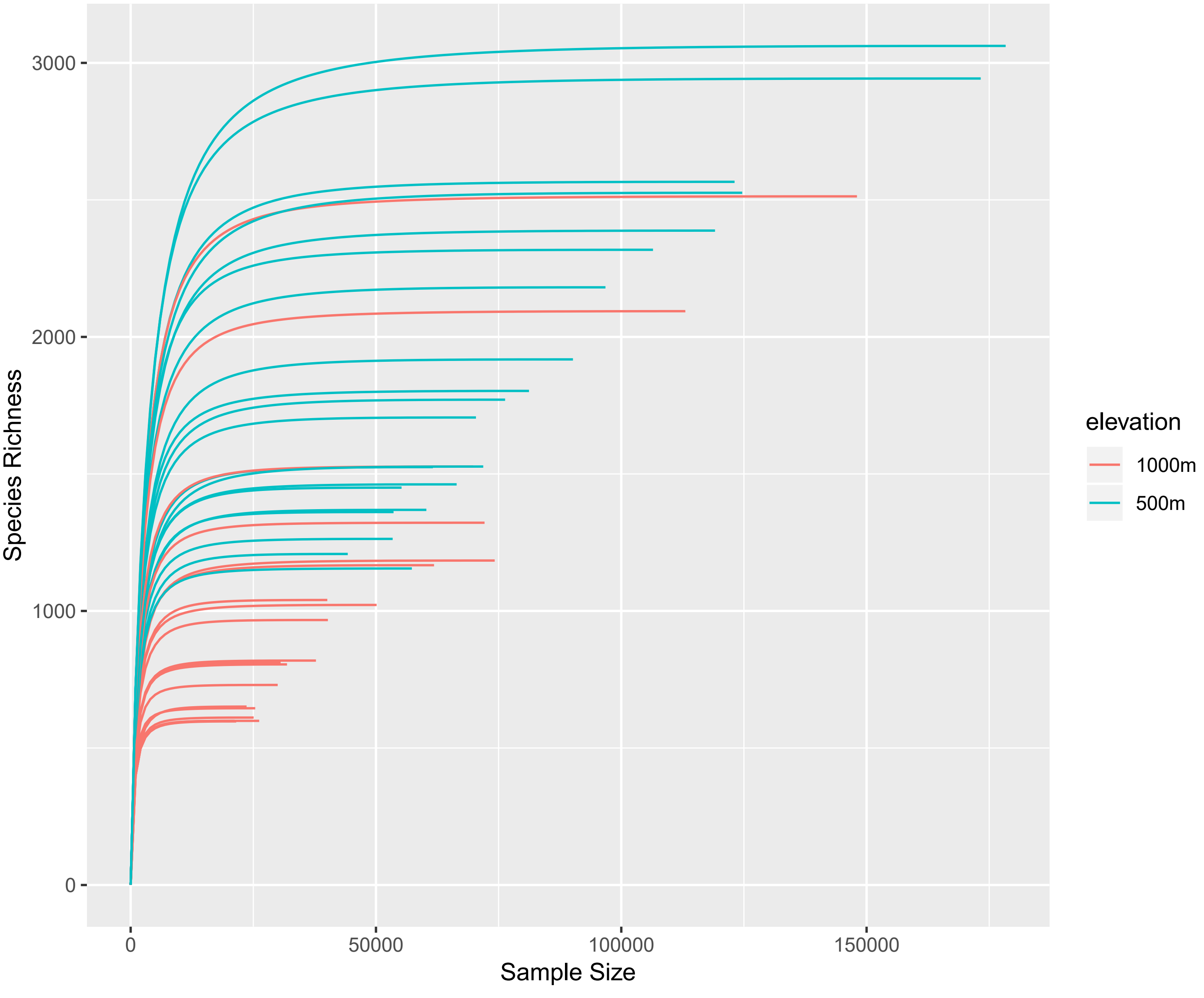

Supplementary figure 1: Rarefaction curves of bacterial reads.

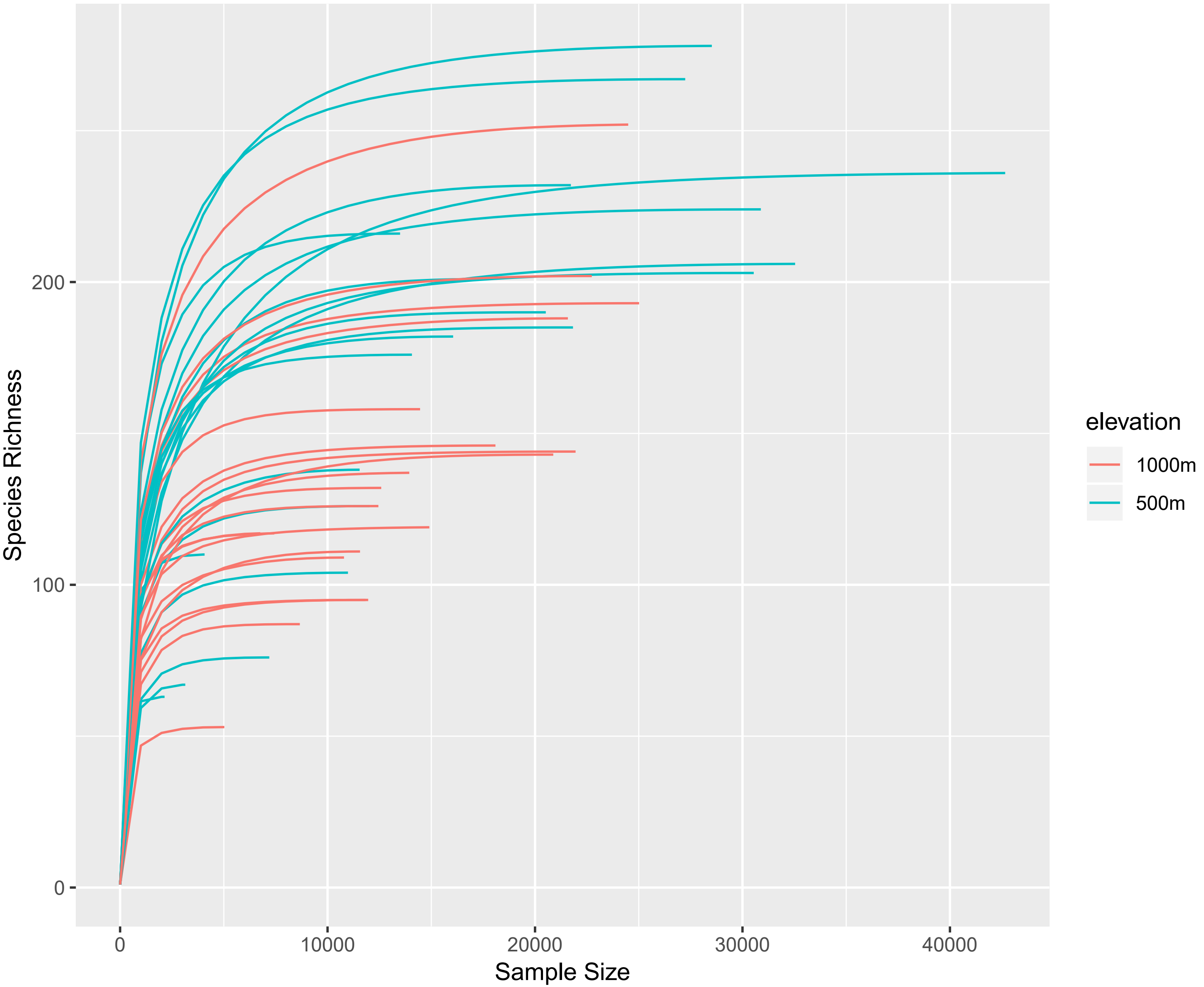

Supplementary figure 2: Rarefaction curves of fungal reads.

Supplementary table 1: List of sample names, index names, and primer sequences used for sequencing.

	sample name	P7 index name	P7 index sequence (5'–>3')	P5 index name	P5 index sequence (5'–>3')
1	bac_1000m_abr_rep3	P7_1	AAGTCGGA	P5_2	TTGACACC
2	fun_1000m_abr_rep3	P7_1	AAGTCGGA	P5_9	AGCAGAAG
3	bac_500m_2/5	P7_1	AAGTCGGA	P5_11	AGTAGGCA
4	fun_500m_2/5	P7_1	AAGTCGGA	P5_12	TGAGTGGA
5	bac_1000m_ctrl_rep5	P7_10	TGCCTCTT	P5_1	AAGTCGGA
6	bac_500m_ctrl_rep1	P7_10	TGCCTCTT	P5_7	GTACAGAG
7	fun_1000m_ctrl_rep5	P7_10	TGCCTCTT	P5_8	TGGACGTT
8	fun_500m_ctrl_rep1	P7_10	TGCCTCTT	P5_10	TGCCTCTT
9	bac_1000m_3/5	P7_10	TGCCTCTT	P5_11	AGTAGGCA
10	fun_1000m_3/5	P7_10	TGCCTCTT	P5_12	TGAGTGGA
11	bac_1000m_grad_rep3	P7_11	AGTAGGCA	P5_1	AAGTCGGA
12	bac_500m_ctrl_rep4	P7_11	AGTAGGCA	P5_7	GTACAGAG
13	fun_1000m_grad_rep3	P7_11	AGTAGGCA	P5_8	TGGACGTT
14	fun_500m_ctrl_rep4	P7_11	AGTAGGCA	P5_10	TGCCTCTT
15	bac_1000m_grad_rep2	P7_12	TGAGTGGA	P5_1	AAGTCGGA
16	bac_500m_ctrl_rep3	P7_12	TGAGTGGA	P5_7	GTACAGAG
17	fun_1000m_grad_rep2	P7_12	TGAGTGGA	P5_8	TGGACGTT
18	fun_500m_ctrl_rep3	P7_12	TGAGTGGA	P5_10	TGCCTCTT
19	bac_1000m_grad_rep4	P7_13	ACCTGGTA	P5_1	AAGTCGGA
20	bac_500m_ctrl_rep5	P7_13	ACCTGGTA	P5_2	TTGACACC
21	bac_500m_ctrl_rep2	P7_13	ACCTGGTA	P5_7	GTACAGAG
22	fun_1000m_grad_rep4	P7_13	ACCTGGTA	P5_8	TGGACGTT
23	fun_500m_ctrl_rep5	P7_13	ACCTGGTA	P5_9	AGCAGAAG
24	fun_500m_ctrl_rep2	P7_13	ACCTGGTA	P5_10	TGCCTCTT
25	bac_1000m_abr_rep1	P7_14	TCGGTAGT	P5_1	AAGTCGGA
26	fun_1000m_abr_rep1	P7_14	TCGGTAGT	P5_8	TGGACGTT
27	bac_1000m_abr_rep2	P7_15	ATAGGAGC	P5_1	AAGTCGGA
28	fun_1000m_abr_rep2	P7_15	ATAGGAGC	P5_8	TGGACGTT
29	bac_1000m_abr_rep5	P7_17	ATCCGTCT	P5_2	TTGACACC
30	fun_1000m_abr_rep5	P7_17	ATCCGTCT	P5_9	AGCAGAAG
31	bac_500m_3/5	P7_17	ATCCGTCT	P5_11	AGTAGGCA
32	fun_500m_3/5	P7_17	ATCCGTCT	P5_12	TGAGTGGA
33	bac_1000m_ctrl_rep1	P7_2	TTGACACC	P5_1	AAGTCGGA
34	bac_1000m_abr_rep4	P7_2	TTGACACC	P5_2	TTGACACC
35	fun_1000m_ctrl_rep1	P7_2	TTGACACC	P5_8	TGGACGTT
36	fun_1000m_abr_rep4	P7_2	TTGACACC	P5_9	AGCAGAAG
37	bac_500m_1/5	P7_2	TTGACACC	P5_11	AGTAGGCA
38	fun_500m_1/5	P7_2	TTGACACC	P5_12	TGAGTGGA
39	bac_500m_abr_rep3	P7_4	TGTTAGGC	P5_2	TTGACACC
40	fun_500m_abr_rep3	P7_4	TGTTAGGC	P5_9	AGCAGAAG

41	bac_500m_5/5	P7_4	TGTTAGGC	P5_11	AGTAGGCA
42	fun_500m_5/5	P7_4	TGTTAGGC	P5_12	TGAGTGGA
43	bac_1000m_ctrl_rep4	P7_5	ACGAGAGA	P5_1	AAGTCGGA
44	bac_500m_abr_rep1	P7_5	ACGAGAGA	P5_2	TTGACACC
45	bac_500m_grad_rep5	P7_5	ACGAGAGA	P5_7	GTACAGAG
46	fun_1000m_ctrl_rep4	P7_5	ACGAGAGA	P5_8	TGGACGTT
47	fun_500m_abr_rep1	P7_5	ACGAGAGA	P5_9	AGCAGAAG
48	fun_500m_grad_rep5	P7_5	ACGAGAGA	P5_10	TGCCTCTT
49	bac_500m_4/5	P7_5	ACGAGAGA	P5_11	AGTAGGCA
50	fun_500m_4/5	P7_5	ACGAGAGA	P5_12	TGAGTGGA
51	bac_1000m_ctrl_rep2	P7_6	TGGTGCAT	P5_1	AAGTCGGA
52	bac_500m_abr_rep5	P7_6	TGGTGCAT	P5_2	TTGACACC
53	bac_500m_grad_rep1	P7_6	TGGTGCAT	P5_7	GTACAGAG
54	fun_1000m_ctrl_rep2	P7_6	TGGTGCAT	P5_8	TGGACGTT
55	fun_500m_abr_rep5	P7_6	TGGTGCAT	P5_9	AGCAGAAG
56	fun_500m_grad_rep1	P7_6	TGGTGCAT	P5_10	TGCCTCTT
57	bac_1000m_5/5	P7_6	TGGTGCAT	P5_11	AGTAGGCA
58	fun_1000m_5/5	P7_6	TGGTGCAT	P5_12	TGAGTGGA
59	bac_1000m_ctrl_rep3	P7_7	GTACAGAG	P5_1	AAGTCGGA
60	bac_500m_grad_rep4	P7_7	GTACAGAG	P5_7	GTACAGAG
61	fun_1000m_ctrl_rep3	P7_7	GTACAGAG	P5_8	TGGACGTT
62	fun_500m_grad_rep4	P7_7	GTACAGAG	P5_10	TGCCTCTT
63	bac_1000m_1/5	P7_7	GTACAGAG	P5_11	AGTAGGCA
64	fun_1000m_1/5	P7_7	GTACAGAG	P5_12	TGAGTGGA
65	bac_1000m_grad_rep1	P7_8	TGGACGTT	P5_1	AAGTCGGA
66	bac_500m_abr_rep2	P7_8	TGGACGTT	P5_2	TTGACACC
67	bac_500m_grad_rep3	P7_8	TGGACGTT	P5_7	GTACAGAG
68	fun_1000m_grad_rep1	P7_8	TGGACGTT	P5_8	TGGACGTT
69	fun_500m_abr_rep2	P7_8	TGGACGTT	P5_9	AGCAGAAG
70	fun_500m_grad_rep3	P7_8	TGGACGTT	P5_10	TGCCTCTT
71	bac_1000m_4/5	P7_8	TGGACGTT	P5_11	AGTAGGCA
72	fun_1000m_4/5	P7_8	TGGACGTT	P5_12	TGAGTGGA
73	bac_1000m_grad_rep5	P7_9	AGCAGAAG	P5_1	AAGTCGGA
74	bac_500m_abr_rep4	P7_9	AGCAGAAG	P5_2	TTGACACC
75	bac_500m_grad_rep2	P7_9	AGCAGAAG	P5_7	GTACAGAG
76	fun_1000m_grad_rep5	P7_9	AGCAGAAG	P5_8	TGGACGTT
77	fun_500m_abr_rep4	P7_9	AGCAGAAG	P5_9	AGCAGAAG
78	fun_500m_grad_rep2	P7_9	AGCAGAAG	P5_10	TGCCTCTT
79	fun_1000m_2/5	P7_9	AGCAGAAG	P5_12	TGAGTGGA
